# Aggf1 attenuates neuroinflammation and BBB disruption via PI3K/Akt/NF-κB pathway after subarachnoid hemorrhage in rats

**DOI:** 10.1186/s12974-018-1211-8

**Published:** 2018-06-09

**Authors:** Qiquan Zhu, Budbazar Enkhjargal, Lei Huang, Tongyu Zhang, Chengmei Sun, Zhiyi Xie, Pei Wu, Jun Mo, Jiping Tang, Zongyi Xie, John H. Zhang

**Affiliations:** 1 Department of Neurosurgery, The Second Affiliated Hospital, Chongqing Medical University, Chongqing, 400010 China; 2 Department of Physiology and Pharmacology, School of Medicine, Loma Linda University, Loma Linda, CA 92354 USA; 3 Department of Anesthesiology, School of Medicine, Loma Linda University, Loma Linda, CA 92354 USA; 4 Department of Neurosurgery, School of Medicine, Loma Linda University, Loma Linda, CA 92354 USA

**Keywords:** Angiogenic factor with G patch and FHA domains 1, Neuroinflammation, Blood-brain barrier, Subarachnoid hemorrhage

## Abstract

**Background:**

Neuroinflammation and blood-brain barrier (BBB) disruption are two critical mechanisms of subarachnoid hemorrhage (SAH)-induced brain injury, which are closely related to patient prognosis. Recently, angiogenic factor with G-patch and FHA domain 1 (Aggf1) was shown to inhibit inflammatory effect and preserve vascular integrity in non-nervous system diseases. This study aimed to determine whether Aggf1 could attenuate neuroinflammation and preserve BBB integrity after experimental SAH, as well as the underlying mechanisms of its protective roles.

**Methods:**

Two hundred forty-nine male Sprague-Dawley rats were subjected to the endovascular perforation model of SAH. Recombinant human Aggf1 (rh-Aggf1) was administered intravenously via tail vein injection at 1 h after SAH induction. To investigate the underlying neuroprotection mechanism, Aggf1 small interfering RNA (Aggf1 siRNA) and PI3K-specific inhibitor LY294002 were administered through intracerebroventricular (i.c.v.) before SAH induction. SAH grade, neurological score, brain water content, BBB permeability, Western blot, and immunohistochemistry were performed.

**Results:**

Expression of endogenous Aggf1 was markedly increased after SAH. Aggf1 was primarily expressed in endothelial cells and astrocytes, as well as microglia after SAH. Administration of rh-Aggf1 significantly reduced brain water content and BBB permeability, decreased the numbers of infiltrating neutrophils, and activated microglia in the ipsilateral cerebral cortex following SAH. Furthermore, rh-Aggf1 treatment improved both short- and long-term neurological functions after SAH. Meanwhile, exogenous rh-Aggf1 significantly increased the expression of PI3K, p-Akt, VE-cadherin, Occludin, and Claudin-5, as well as decreased the expression of p-NF-κB p65, albumin, myeloperoxidase (MPO), TNF-α, and IL-1β. Conversely, knockdown of endogenous Aggf1 aggravated BBB breakdown, inflammatory response and neurological impairments at 24 h after SAH. Additionally, the protective roles of rh-Aggf1 were abolished by LY294002.

**Conclusions:**

Taken together, exogenous Aggf1 treatment attenuated neuroinflammation and BBB disruption, improved neurological deficits after SAH in rats, at least in part through the PI3K/Akt/NF-κB pathway.

**Electronic supplementary material:**

The online version of this article (10.1186/s12974-018-1211-8) contains supplementary material, which is available to authorized users.

## Background

Aneurysmal subarachnoid hemorrhage (SAH) is a devastating cerebrovascular disease with a high rate of mortality and disability [1]. Neuroinflammation and blood-brain barrier (BBB) disruption have been recognized as important pathological processes in early brain injury (EBI) after SAH [2–4]. Neuroinflammatory responses are characterized by resident microglial and astrocyte activation, infiltration of peripheral leukocytes, and release of pro-inflammatory mediators, which exacerbate BBB disruption that further amplifies inflammatory response and worsens neurological impairments [5]. Therefore, developing a protective strategy against neuroinflammation and BBB disruption may be effective for patients with SAH.

Angiogenic factor with G patch and FHA domains 1 (Aggf1, also known as VG5Q) was initially identified as a vascular endothelium-derived protein and promoted angiogenesis as strongly as vascular endothelial growth factor A (VEGFA) [6]. Mounting evidence suggested that Aggf1 exerts multiple roles in various pathological processes, including embryogenesis, tumorigenesis, cirrhosis, apoptosis, inflammation, and autophagy [7–12]. Aggf1 was proved to reduce the release of inflammatory molecules in myocardial ischemia model [13]. Moreover, an in vivo study revealed that Aggf1 exerts anti-inflammatory property through suppressing TNF-α-induced endothelial activation in a mouse model of hindlimb ischemia [14]. Previous study demonstrated exogenous Aggf1 treatment could increase vessel density and improve blood flow after limb ischemia in mice [15]. Aggf1 maintained vascular integrity by inhibiting VE-cadherin phosphorylation in myocardial ischemia/reperfusion models [16]. Despite the well-established roles for Aggf1 in inflammation and vascular integrity, the effects of Aggf1 on neuroinflammation and BBB integrity after SAH have not been investigated.

The activation of PI3K/Akt/NF-κB pathway is involved in the stabilization of BBB integrity by increasing the expression of tight junction proteins and the alleviation of inflammation via decreasing inflammatory mediators in stroke and neurodegenerative disease [17–19]. Recent studies showed that Aggf1 possessed the protective roles by activating PI3K/Akt pathway in myocardial ischemia-reperfusion injury and in cell cultures [16].

In the present study, we hypothesized that intravenous administration of recombinant human Aggf1 (rh-Aggf1) could attenuate neuroinflammation and BBB disruption after SAH, as well as the protective effects of Aggf1 are mediated through PI3K/Akt/NF-κB pathway.

## Methods

### Animals

All procedures and protocols for this study were approved by the Institutional Animal Care and Use Committee (IACUC) of Loma Linda University in accordance with the guideline for the Care and Use of Laboratory Animals by National Institutes of Health. Two hundred forty-nine male Sprague-Dawley rats (Indianapolis, IN, USA) weighing 300–320 g were used, and they were housed in a light- and temperature-controlled room with unlimited access to food and water.

### SAH model

The endovascular perforation model was induced as previously described [20]. Briefly, rats were intubated and maintained with 3% isoflurane anesthesia in the air. Rodents were placed in a supine position, and the neck was opened with a sharp scalpel in the midline. After localization of the appropriate vessels, a sharpened 3-cm, 4–0 nylon suture was inserted into the left internal carotid artery through the external carotid artery and the common carotid bifurcation. The suture was advanced until resistance was reached, further advanced in order to puncture the vessel, and then immediately withdrawn after artery perforation. The sham-operated group underwent the same procedure without an endovascular puncture. After removal of the suture, the skin incision was sutured and the rats were housed individually in heated cages until recovery.

### Experimental design

**Fig. 1:**
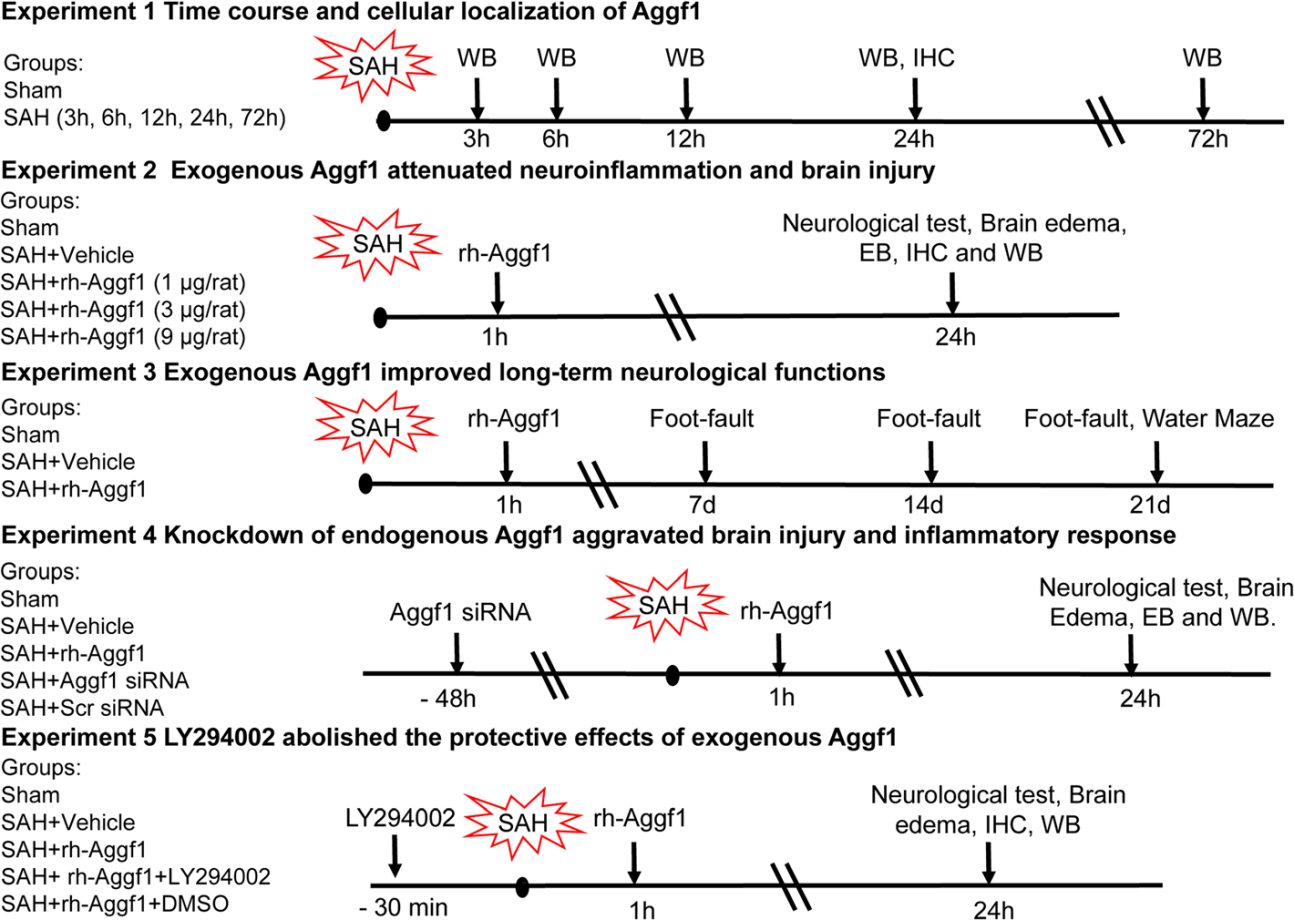
Experimental design and animal groups. Aggf1, angiogenic factor with G patch and FHA domains 1; DMSO, dimethyl sulfoxide; EB, Evans blue; IHC, immunohistochemistry; LY294002, PI3K inhibitor; SAH, subarachnoid hemorrhage; Scr siRNA, scrambled siRNA; WB, Western blot

Animals were randomly assigned to five separate experiments as described (Fig. 1). The experimental group information was blinded to the surgeon and researchers who performed the neurobehavioral tests and Western blot/immunofluorescence as well as data analysis.

#### Experiment 1

The time course of endogenous Aggf1 in the ipsilateral (left) cerebral cortex was measured by Western blot analysis. Double immunofluorescence staining was performed to characterize the cellular localization of Aggf1 at 24 h after SAH.

#### Experiment 2

To assess the effects of recombinant human Aggf1 (rh-Aggf1) on neuroinflammation, three doses of rh-Aggf1 (1, 3, 9 μg/rat; R&D Systems, USA) dissolved in normal saline (NS) were administered intravenously via the tail vein at 1 h after SAH. Neurological scores, SAH grade, and brain water content were measured at 24 h after SAH. Rats were randomly divided into five groups: sham, SAH+vehicle (normal saline, NS), SAH+rh-Aggf1 (1 μg/rat), SAH+rh-Aggf1 (3 μg/rat), SAH+rh-Aggf1 (9 μg/rat). Evans blue (EB) extravasation, neutrophil infiltration, and inflammatory factor molecules expression were evaluated at 24 h after SAH in sham, SAH+vehicle, and SAH+rh-Aggf1 (3 μg/rat).

#### Experiment 3

To evaluate the effects of rh-Aggf1 on long-term neurological functions, the foot-fault test was conducted at the first, second, and third week after SAH, and Morris water maze was performed at days 21–25 post-SAH. Rats were randomly divided into three groups: sham, SAH+vehicle, SAH+rh-Aggf1 (3 μg/rat).

#### Experiment 4

To investigate the effect of in vivo knockdown of endogenous Aggf1 on neuroinflammation, Aggf1 small interfering RNA (Aggf1 siRNA) was administered intracerebroventricularly (i.c.v) at 48 h before SAH induction. Neurological scores, brain water content, EB extravasation, and Western blot were assessed at 24 h after SAH. Rats were randomly assigned to five groups: sham, SAH+vehicle (normal saline, NS), SAH+rh-Aggf1, SAH+Aggf1 siRNA, and SAH+scrambled siRNA (Scr siRNA). Additionally, to validate the knockdown efficiency of Aggf1 siRNA, the expression of Aggf1 in the ipsilateral (left) cerebral cortex was analyzed by Western blot. Rats were randomly divided into four groups: Naive+Scr siRNA, Naive+Aggf1 siRNA, SAH+ Scr siRNA, and SAH+Aggf1 siRNA.

#### Experiment 5

To explore the underlying mechanisms of rh-Aggf1-mediated neuroprotective effects, PI3K-specific inhibitor LY294002 was injected i.c.v. at 30 min before SAH induction and then followed with rh-Aggf1 (3 μg/rat) treatment. Neurobehavioral tests, brain water content, immunofluorescence staining, and Western blot were examined at 24 h after SAH. Rats were randomly assigned to five groups: sham, SAH+vehicle, SAH+rh-Aggf1, SAH+rh-Aggf1+LY294002, and SAH+rh-Aggf1+dimethyl sulfoxide (DMSO).

### SAH grading

The assessment of SAH grading score was performed at 24 h after SAH by an independent investigator blinded to the experimental group information as previously described [21]. Briefly, the basal cistern was divided into six segments that were scored from 0 to 3 according to the amounts of subarachnoid blood. Rats with the grade < 8 at 24 h after SAH were excluded from this study.

### Short-term neurological function evaluation

The neurological status was assessed at 24 h after SAH using modified Garcia and beam balance as previously described [22]. The modified Garcia test (maximum score = 18) included vibrissae touch, trunk touch, spontaneous activity, spontaneous movement of the four limbs, forelimbs outstretching, and climbing capacity. The beam balance test (maximum score = 4) was conducted to assess the ability of rats to walk on a wooden beam for 1 min. High scores indicated better neurological function. The neurological assessment was performed by an investigator blind to experiment.

### Long-term neurological score evaluation

The foot-fault test was measured to assess the motor-sensory deficits by a partner blinded to the group information as previously described [23]. Briefly, rats were placed on a horizontal grid floor for 2 min. The foot-fault was defined as when the rat incorrectly placed a fore- or hindlimb, and it fell through one of the openings in the grid. The number of foot-fault was recorded and used for the statistical analysis.

The Morris water maze was performed to assess the spatial learning memory by an independent researcher blinded to the experimental group information as previously described [24, 25]. Briefly, rats were placed using a semi-random set of start locations to find a visible platform above the water level in 60 s. After that, the rats were guided to stay on the platform for 5 s. At the last day, the probe trial was performed in which the rats were allowed to swim to search the platform submerged in the water. Swim path, swim distance, escape latency, and probe quadrant duration were recorded by a computerized tracking system (Noldus Ethovision; Noldus, Tacoma, WA, USA).

### Brain water content

Brain samples were collected at 24 h after surgery and separated into the left hemisphere, right hemisphere, cerebellum, and brain stem. Each part was weighed immediately after removal (wet weight) and then dried in an oven at 105 °C for 72 h (dry weight). After that, the percentage of brain water content was calculated as [(wet weight − dry weight)/wet weight] × 100%.

### Blood-brain barrier permeability

BBB permeability was evaluated by EB extravasation using spectrophotometry as previously described [20]. A 2% solution of EB in normal saline (5 ml/kg of body weight) was injected into the right femoral vein at 24 h after SAH. The stain was allowed to circulate for an additional 60 min before sacrifice. Then the rats were transcardially perfused with 120 ml of ice-cold PBS in deep anesthesia. The collected brain samples were subsequently removed and divided into four parts: right hemisphere, left hemispheres, cerebellar, and brain stem and stored in a − 80 °C freezer. After homogenization of each sample with PBS, the solution was centrifuged for 30 min at 14,000 r/min in 4 °C. The supernatant was collected, mixed with equal amount of trichloroacetic acid, and incubated at room temperature for 1 h. Then, the sample was centrifuged for 30 min at 15,000 r/min in 4 °C to separate the supernatant for measurements. EB stain results were measured by a spectrophotometer (Thermo Spectronic Genesys 10 UV, Thermo Fischer Scientific Inc., Waltham, MA, USA) at 610 nm and quantified with a standard curve. The results are presented as fold increase compared to sham group.

### Intracerebroventricular administration

Intracerebroventricular drug administration was performed as previously described [24]. Briefly, rats were placed in a stereotaxic apparatus under anesthesia with 2.5% isoflurane in 70/30% medical air/oxygen. The needle of a 10-μl Hamilton syringe (Microliter701; Hamilton Company, Reno, NV, USA) was inserted into the right lateral ventricle through a burr hole using the following coordinates relative to bregma: 1.5 mm posterior, 0.9 mm lateral, and 3.3 mm below the horizontal plane of the bregma. For Aggf1 in vivo knockdown, two different rat Aggf1 siRNA duplexes (Thermo Fisher Scientific, Waltham, MA) (total 500 pmol) were dissolved in RNase free suspension buffer and then infused into the right lateral ventricle via a pump with the rate 1 μl/min at 48 h before SAH induction. The same volume of Scr siRNA (Thermo Fisher Scientific, Waltham, MA) was used as a negative control. PI3K-specific inhibitor LY294002 (Selleck Chemicals, Houston, USA) was prepared at 50 mmol/l in PBS (contains 25% DMSO) with a total volume 5 μl. ICV injection was performed. The same volume of DMSO was used as a negative control. After injection, the needle was kept in place for an additional 5 min and retracted slowly. Then, the burr hole was sealed with bone wax immediately, and the rats were allowed to recover after sutures.

### Western blot

Western blot analysis was performed as previously described [26]. After sample preparation, equal amounts of a sample protein (50 μg) were loaded onto an SDS-PAGE gel. First, electrophoresis and transfer of the samples to a nitrocellulose membrane were performed. Second, the membrane was blocked for 2 h at room temperature and incubated overnight at 4 °C with the following primary antibodies: anti-Aggf1 (1:2000, Sigma-Aldrich, USA), anti-albumin (1:5000, Abcam, USA), anti-Claudin-5 (1:1000, Abcam, USA), anti-myeloperoxidase (MPO, 1:1000, Abcam, USA), anti-Occludin (1:2000, Abcam, USA), anti-PI3K (1:1000, Cell signaling, USA), anti-Akt (1:1000, Cell signaling, USA), anti-phospho-Akt (p-Akt, 1:1000, Cell signaling, USA), anti-NF-κB p65 (1:500, Santa Cruz, USA), anti-phospho-NF-κB p65 (p-NF-κB p65, 1:500, Santa Cruz, USA), anti-VE-cadherin (1:500, Santa Cruz, USA), anti-IL-1β (1:300, Santa Cruz, USA), anti-TNF-a (1:300, Santa Cruz, USA), and anti-β-actin (1:5000, Santa Cruz, USA). Appropriate secondary antibodies (1:5000, Santa Cruz, USA) were selected to incubate with the membrane for 2 h at room temperature. Then, blot bands were visualized with an ECL reagent (Amersham Biosciences UK Ltd., PA, USA). Non-saturated bands were selected to perform densitometry quantification using Image J software (Image J 1.4, NIH, USA).

### Immunofluorescence staining

Double fluorescence staining was conducted as described previously [27]. The rats were deeply anesthetized at 24 h post-SAH and were transcardially perfused with 60-ml ice-cold PBS followed by 60 ml of 10% paraformaldehyde. The whole brains were collected and then fixed in 10% paraformaldehyde for 24 h followed by 30% sucrose solution for another 72 h. After being frozen at − 80 °C, the brain was cut into 10-μm-thick coronal sections using a cryostat (LM3050S; Leica Microsystems, Bannockburn, III, Germany). To perform double immunohistochemistry staining, the brain sections were incubated with primary antibody of anti-neuronal nuclei (NeuN, 1:200, Abcam, USA), anti-glial fibrillary acidic protein (GFAP, 1:500, Abcam, USA), anti-ionized calcium-binding adaptor molecule 1 (Iba-1, 1:200, Abcam, USA), anti-von Willebrand factor (vWF, 1:100, FITC, Abcam, USA), anti-myeloperoxidase (MPO, 1:200, Santa Cruz, USA), and anti-Aggf1 (1:100, Sigma-Aldrich, USA) overnight at 4 °C. After being incubated with the appropriate secondary antibody (1:200, Jackson Immunoresearch, West Grove, PA, USA) at room temperature for 2 h, the sections were visualized and photographed with a fluorescence microscope (Leica Microsystems, Germany). Microphotographs were analyzed with LASX software. The numbers of Iba-1-positive cells and MPO-positive cells were identified and counted in three different fields in the ipsilateral cortex from five random coronal sections per brain, and data were expressed as cells/field [22].

### Statistical analysis

The sample size was calculated based on extensive review of the literature, our previous preclinical studies in the same rat model of SAH, and preliminary data collected for the treatment efficacy of rh-Aggf1 against SAH. We utilized the mean difference between the groups, the observed standard deviations from previous SAH studies and preliminary data, a power of 0.8, and an alpha of 0.05 on a two-sided test in a sample size calculator (SigmaPlot) to determine the animal numbers. We have identified that we need an *n* = 6/group to reach statistical significance for the measurements.

All statistical analyses were performed using GraphPad Prism for Windows (Graph Pad Software Inc., San Diego, CA, USA). Data were represented as mean ± SD. Statistical differences among groups were analyzed by using one-way ANOVA followed by Turkey post hoc test. *P* value of < 0.05 was considered statistically significant.

## Results

### Mortality and exclusion

The overall mortality of SAH rats was 16.36% (35/214); no rats died in the sham group. According to the SAH grading score, 13 rats were excluded from this study due to low-grade SAH (Additional file 1: Table S1). Subarachnoid blood clots were markedly shown around the circle of Willis (Additional file 2: Figure S1A). There was no statistical difference in SAH grading scores among the SAH groups (Additional file 2: Figure S1B).

### Time course and spatial expression of Aggf1 after SAH

**Fig. 2:**
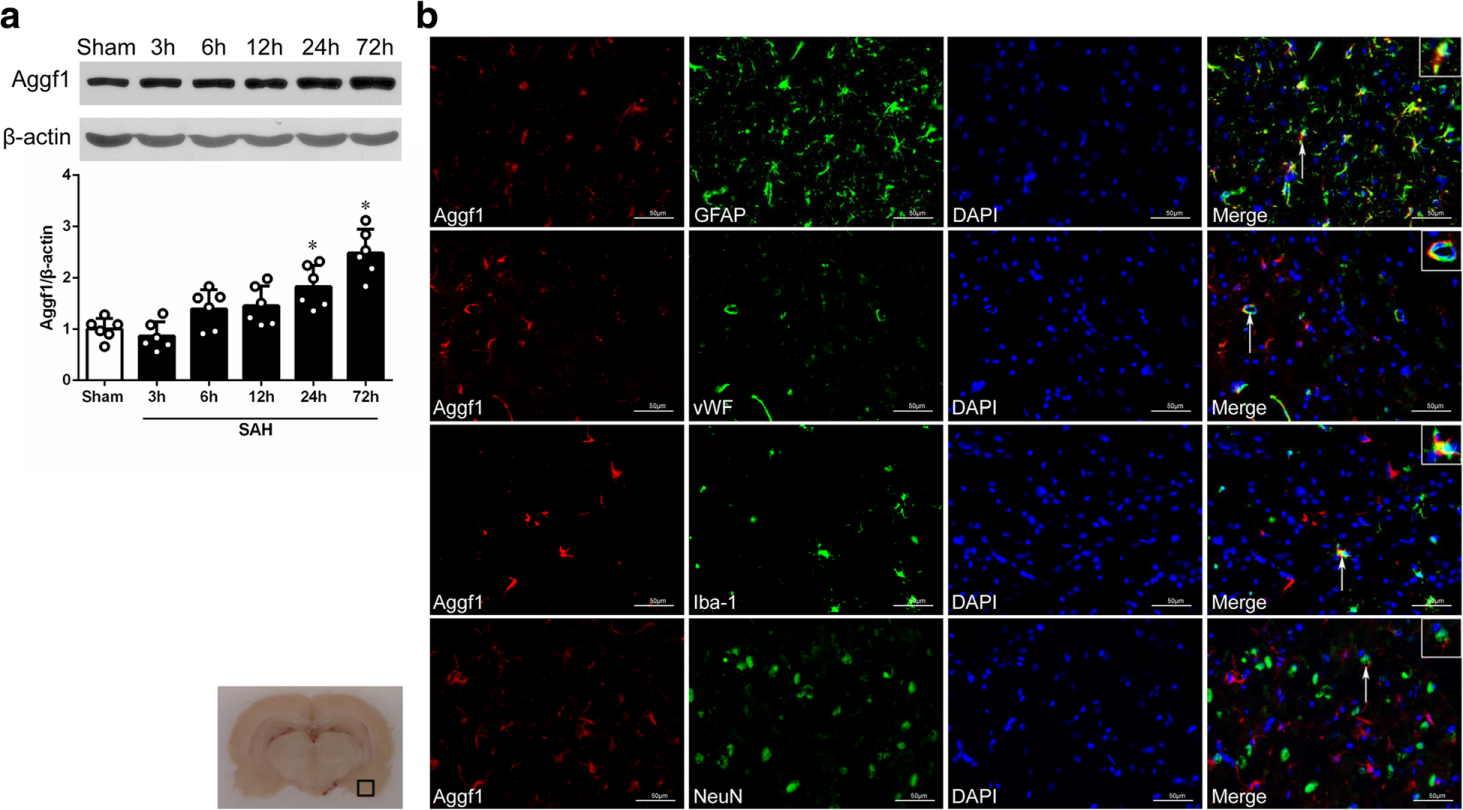
Expression of angiogenic factor with G patch and FHA domains 1 (Aggf1) after subarachnoid hemorrhage (SAH). **a** Representative Western blot band and densitometric quantification of time-dependent expression of Aggf1 after SAH. The expression of Aggf1 was upregulated at 24 h and peaked at 72 h after SAH. *P < 0.05 vs sham. Data were presented as mean ± SD, *n* = 6 per group. **b** Colocalization of Aggf1 with astrocyte (GFAP), endothelium (vWF, green), and microglia (Iba-1) at 24 h after SAH. Nuclei are stained with DAPI (blue). Left panel indicates the location of staining in the brain (small black box), *n* = 3 per group, scale bar = 50 μm

The expression of endogenous Aggf1 in the ipsilateral (left) cerebral cortex was assessed by Western blots. As shown in Fig. 2a, there was a significant increase of Aggf1 level at 24 h, which peaked at 72 h after SAH when compared to the sham group. Double immunofluorescence staining revealed that Aggf1 was mainly expressed in the endothelial cells and astrocytes, as well as microglia in the ipsilateral basal cortex at 24 h after SAH (Fig. 2b).

### Rh-Aggf1 treatment improved short-term neurobehavioral functions and reduced brain edema and BBB permeability after SAH

**Fig. 3:**
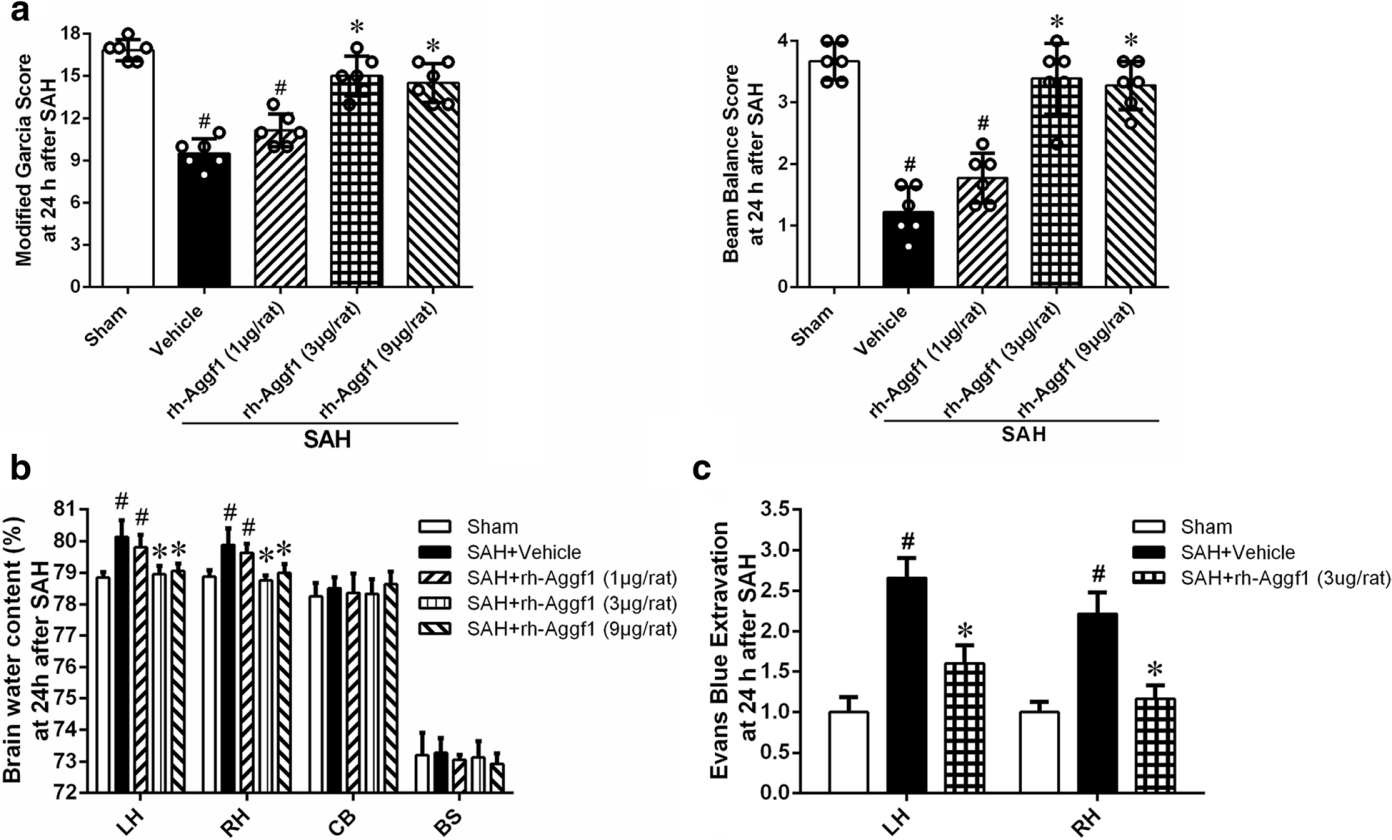
Exogenous Aggf1 attenuated neurological deficits, BBB edema, and BBB permeability. **a**, **b** Treatment with rh-Aggf1 improved neurological deficits and reduced brain edema at 24 h after SAH. ^#^*P* < 0.05 vs sham; *P < 0.05 vs vehicle and rh-Aggf1 (1 μg/rat), *n* = 6 per group. **c** Administration of rh-Aggf1 decreased EB extravasation at 24 h after SAH. ^#^*P* < 0.05 vs sham; *P < 0.05 vs vehicle, *n* = 6 per group. EB, Evans blue

The neurological scores of modified Garcia and beam balance were significantly reduced at 24 h after SAH in the SAH+vehicle and SAH+rh-Aggf1 (1 μg/rat) groups. However, administration of rh-Aggf1 (3 μg/rat) and rh-Aggf1 (9 μg/rat) significantly improved the neurological deficits (Fig. 3a). SAH induction significantly increased the brain edema of both hemispheres in the SAH+vehicle and SAH+rh-Aggf1 (1 μg/rat) groups compared to the sham group at 24 h after SAH (Fig. 3b). Administration of rh-Aggf1 (3 μg/rat) and rh-Aggf1 (9 μg/rat) considerably reduced brain edema (Fig. 3b). Based on these results, the optimal dose of rh-Aggf1 was 3 μg/rat, which was used for the rest of the experiments.

BBB permeability was evaluated by EB extravasation in both cerebral hemispheres. Although EB extravasation in the SAH+vehicle group was markedly increased at 24 h after SAH, rh-Aggf1 treatment (3 μg/rat) significantly decreased EB dye leakage in both hemispheres compared with the SAH+vehicle group (Fig. 3c).

### Exogenous Aggf1 alleviated neutrophil infiltration and IL-1β, TNF-α expression

**Fig. 4:**
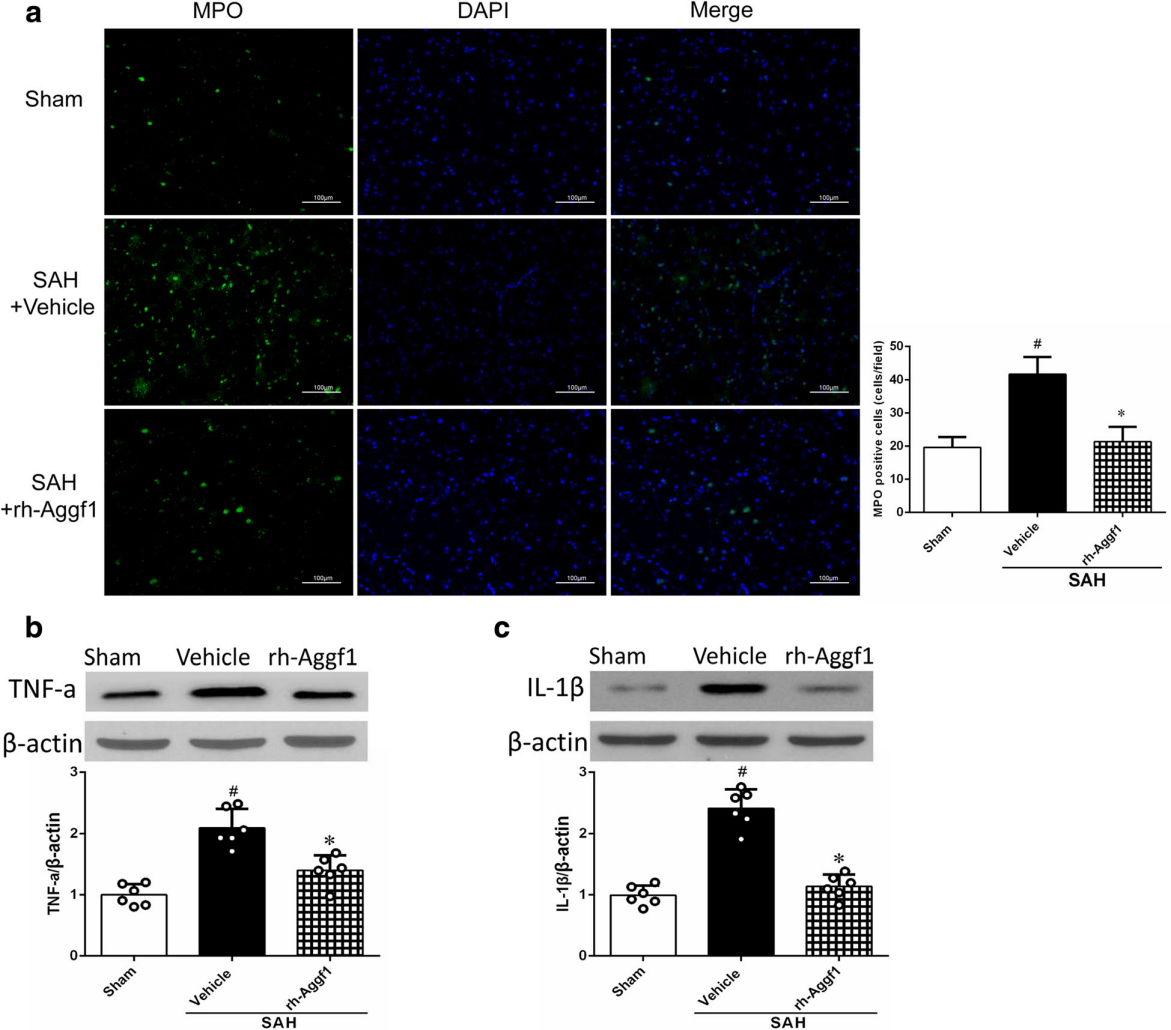
Exogenous Aggf1 alleviated neutrophil infiltration and inflammatory molecule expression at 24 h subarachnoid hemorrhage (SAH). **a** Immunofluorescence revealed that treatment with rh-Aggf1 reduced the number of MPO-positive cells in the ipsilateral cortex, *n* = 3 per group, scale bar = 100 μm. **b**, **c** Relative fold changes of inflammatory cytokines (TNF-α and IL-1β) after exogenous Aggf1 treatment, *n* = 6. ^#^*P* < 0.05 vs sham; *P < 0.05 vs vehicle. MPO, myeloperoxidase

MPO activity, an indicator of neutrophil accumulation, was evaluated in our study. As shown in Fig. 4a, significant increase of neutrophil accumulation was observed in the SAH+vehicle group. However, treatment with rh-Aggf1 significantly reduced the extent of neutrophil accumulation. In addition, the expression of TNF-α and IL-1β were increased in the SAH+vehicle group, while administration of exogenous rh-Aggf1 inhibited TNF-α and IL-1β expression (Fig. 4b, c).

### Exogenous Aggf1 improved long-term neurological functions after SAH

**Fig. 5:**
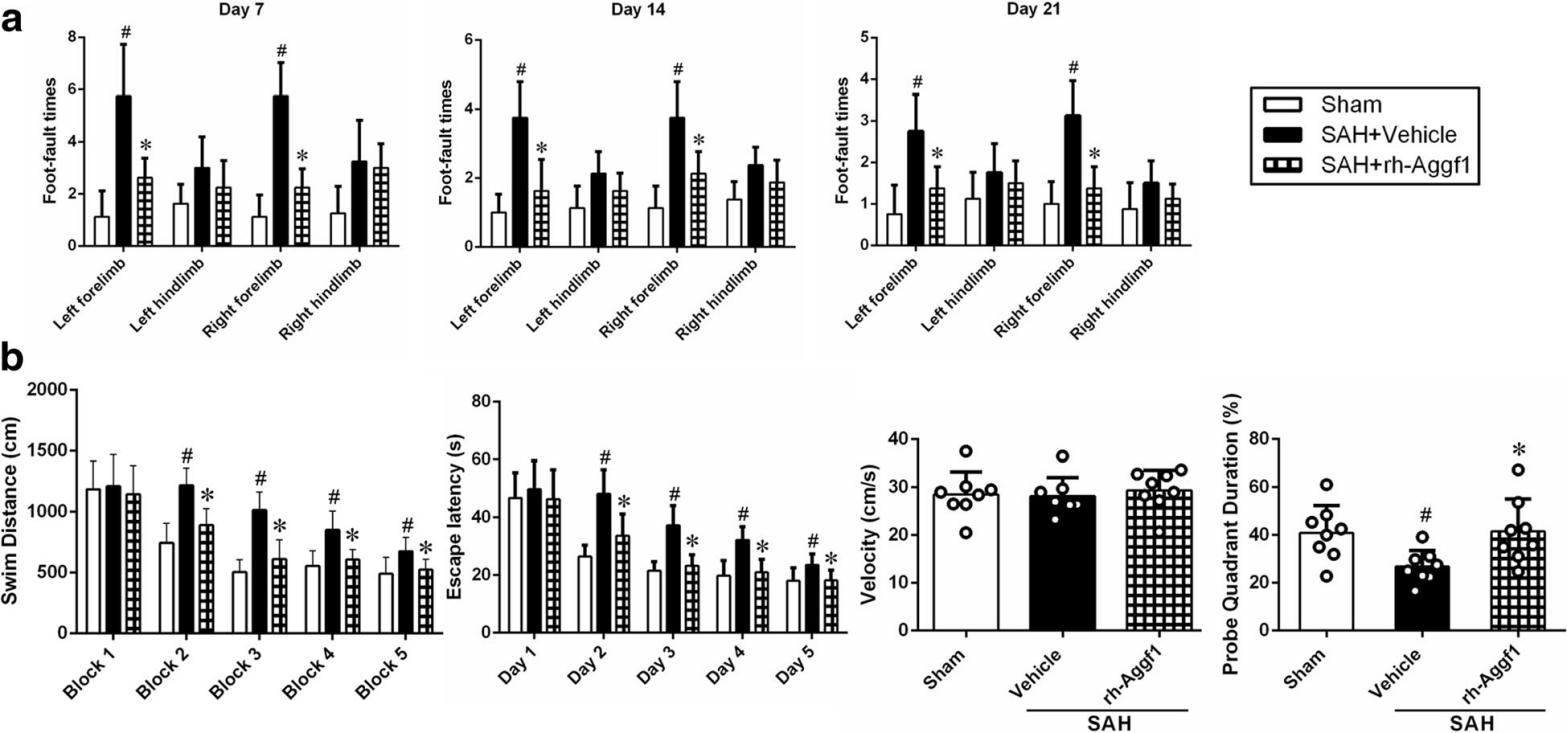
Exogenous Aggf1 improved long-term neurological functions. **a** rh-Aggf1 treatment decreased foot-fault times at days 7, 14, and 21 after subarachnoid hemorrhage (SAH). **b** Administration of rh-Aggf1 improved water maze performances on days 22–25 after SAH. ^#^*P* < 0.05 vs sham; *P < 0.05 vs vehicle, *n* = 8 per group

The rats in the SAH+vehicle group showed significant neurological deficits in foot-faults both in the left forelimb and right forelimb than in the sham group at days 7, 14, and 21 after SAH. However, administration of rh-Aggf1 significantly improved the neurological deficits in foot-fault test both in the left forelimb and right forelimb (Fig. 5a). In the water maze test, the travel distance and escape latency for the rats to find the platform were significantly increased in the SAH+vehicle group when compared to the sham group. However, a significantly shorter distance on block 2, 3, 4, and 5 and a decrease in time to find the platform at days 22, 23, 24, and 25 were observed in the SAH+rh-Aggf1 group (Fig. 5b). In probe quadrant trial, the rats in the SAH+vehicle group spent remarkably less time in the target quadrant when compared to the sham group, while the reference memory deficits were significantly improved with rh-Aggf1 treatment (Fig. 5b). There was no significant difference in swimming velocity among all three groups.

### Knockdown of endogenous Aggf1 exacerbated brain injury and inflammatory response after SAH

**Fig. 6:**
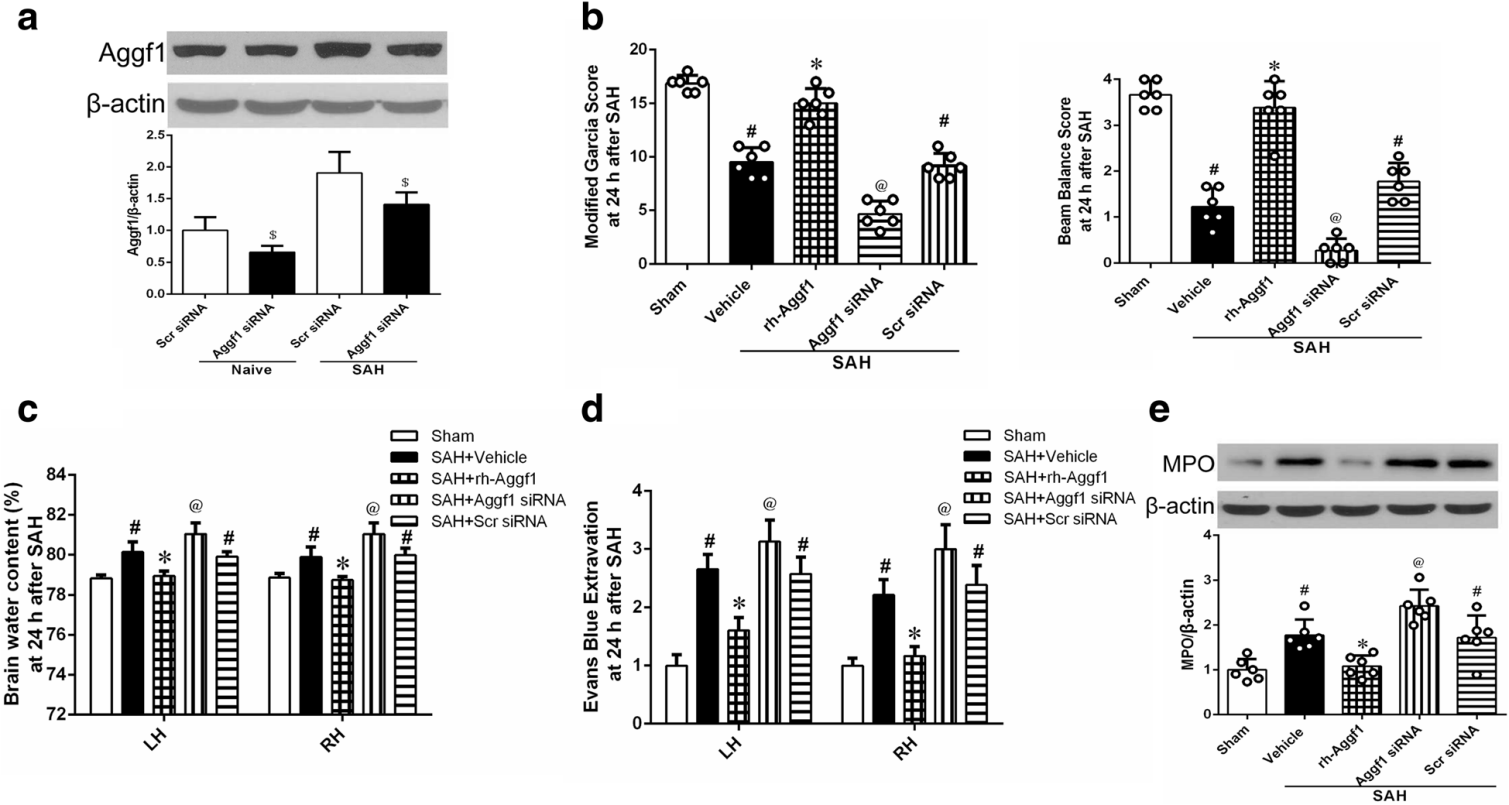
The effect of Aggf1 siRNA on neurological functions, brain water content, BBB permeability, and inflammatory response at 24 h after subarachnoid hemorrhage (SAH). **a** The expression of Aggf1 was significantly reduced in the ipsilateral cortex by Aggf1 siRNA at 24 h after SAH. ^$^*P* < 0.05 vs Scr siRNA. **b**–**e** Knockdown of Aggf1 using Aggf1 siRNA aggravated neurological impairments and increased brain edema and EB extravasation and MPO expression at 24 h following SAH. ^#^*P* < 0.05 vs sham; *P < 0.05 vs vehicle; ^@^*P* < 0.05 vs vehicle, rh-Aggf1, and Scr siRNA. EB, Evans blue; MPO, myeloperoxidase; Scr siRNA, scrambled siRNA

To further verify the protective role of Aggf1 post-SAH, Aggf1 siRNA was injected i.c.v. in order to silence endogenous Aggf1. Western blot showed that the Aggf1 expression was inhibited by Aggf1 siRNA at 72 h after injection (Fig. 6a). The knockdown of Aggf1 exacerbated neurological deficits (Fig. 6b) and increased brain edema (Fig. 6c) and BBB permeability (Fig. 6d) at 24 h post-SAH. Furthermore, the knockdown of Aggf1 increased MPO expression (Fig. 6e).

### Effect of rh-Aggf1 on the expression of downstream molecules of PI3K/Akt/NF- κB signaling pathway

**Fig. 7:**
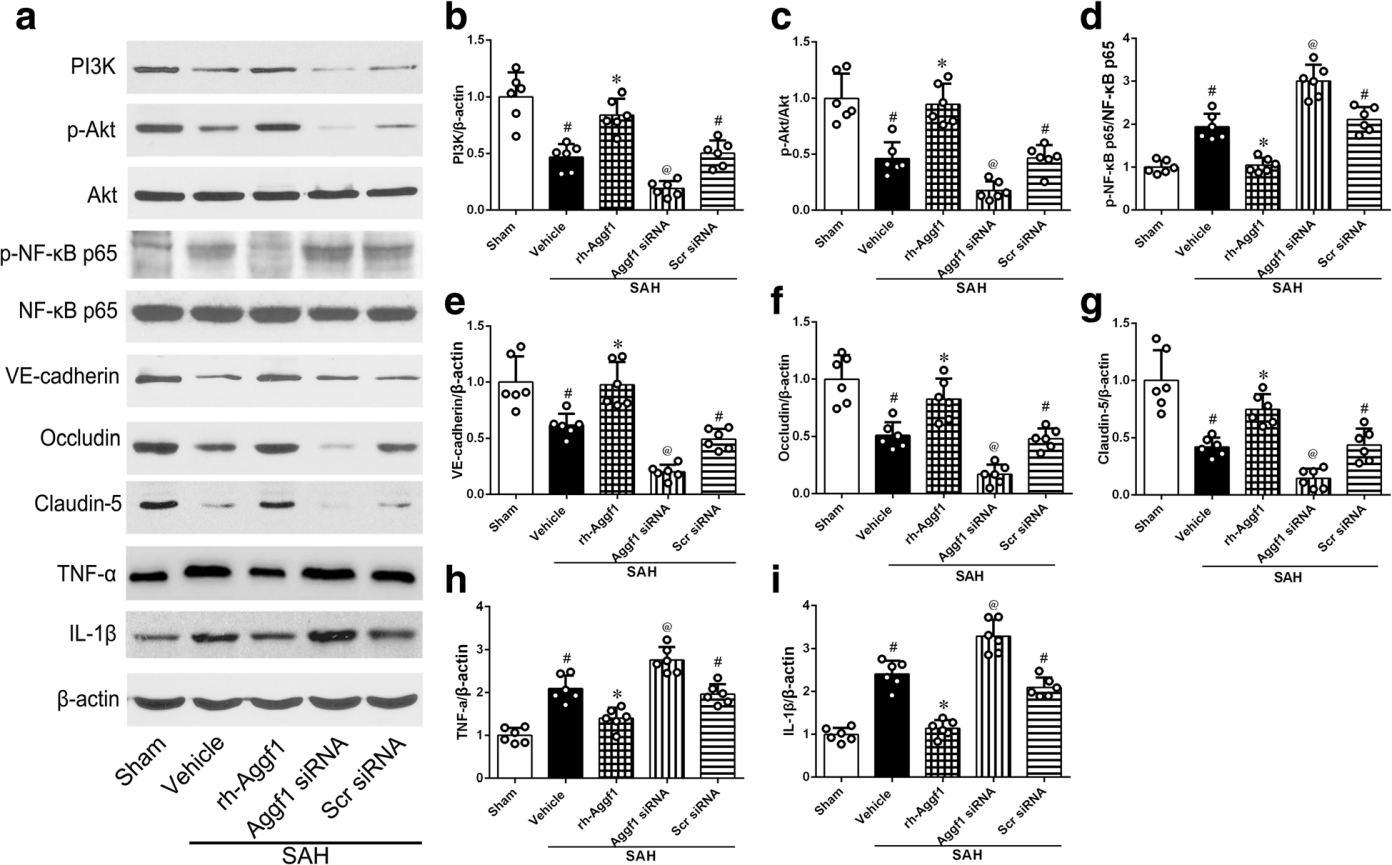
The effect of Aggf1 treatment on expression of downstream molecules at 24 h after subarachnoid hemorrhage (SAH). **a** Representative Western blot band of the downstream signaling pathway protein. **b**–**i** Densitometric quantification suggested that exogenous Aggf1 significantly upregulated the levels of PI3K, p-Akt, VE-cadherin, Occludin, and claudin-5, which were markedly reduced at 24 h post-SAH. Moreover, treatment with rh-Aggf1 significantly decreased p-NF-κB p65, TNF-α, and IL-1β, at the same time. Conversely, knockdown of endogenous Aggf1 using Aggf1 siRNA resulted in a decrease of PI3K, p-Akt, VE-cadherin, Occludin, and claudin-5 and an increase of p-NF-κB p65, TNF-α, and IL-1β. ^#^*P* < 0.05 vs sham; *P < 0.05 vs vehicle; ^@^*P* < 0.05 vs rh-Aggf1, vehicle, and Scr siRNA. Scr siRNA, scrambled siRNA

SAH decreased the expression of PI3K and p-Akt compared with the sham group, whereas rh-Aggf1 significantly increased the levels of PI3K, p-Akt, Occludin, Claudin-5, and VE-cadherin and decreased the expression of p-NF-κB p65, IL-1β, and TNF-α. By contrast, knockdown of Aggf1 using Aggf1 siRNA got opposite changes on the expression of downstream signaling molecules compared with the rh-Aggf1 treatment group (Fig. 7a–i).

### Exogenous Aggf1 treatment inhibited microglia activation after SAH

**Fig. 8:**
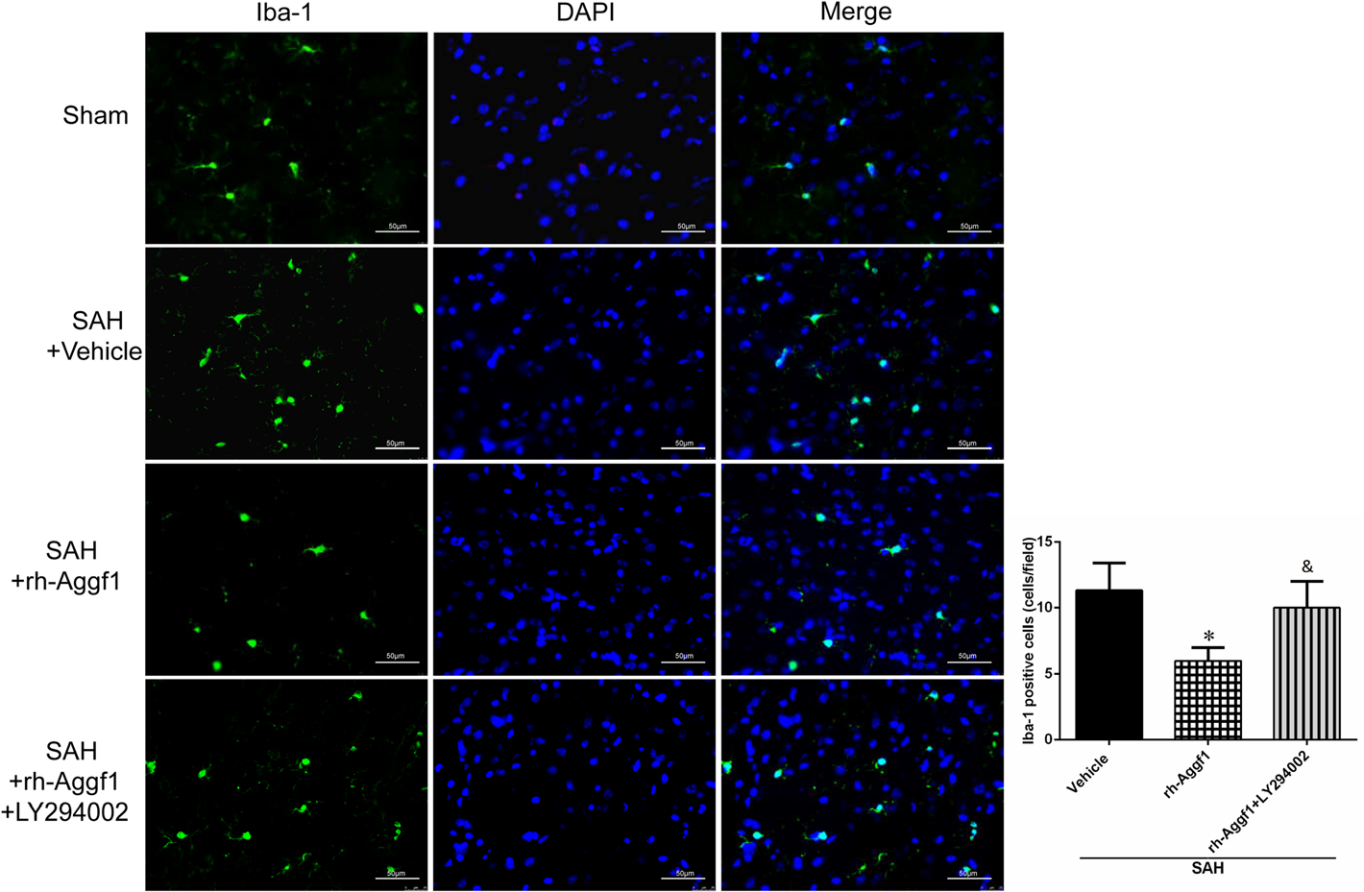
Microglia activation in the ipsilateral cortex at 24 h after subarachnoid hemorrhage (SAH). Representative images and quantification of ionized calcium binding adaptor molecule 1 (Iba-1)-stained microglia showed that rh-Aggf1 treatment reduced the number of Iba-1-positive cells, while this effect was reversed by LY294002. *P < 0.05 vs vehicle; ^&^*P* < 0.05 vs rh-Aggf1. *n* = 3 per group, scale bar = 50 μm

We assessed whether the anti-inflammatory effect of rh-Aggf1 was related to a reduction of microglia activation in the ipsilateral cortex. As shown in Fig. 8, the numbers of Iba-1-positive cells were significantly increased in the ipsilateral cortex in the SAH+vehicle group at 24 h after SAH, and activated microglia showed shorter processes. Administration of rh-Aggf1 dramatically reduced the number of Iba-1-positive cells, while this effect was reversed by PI3K-specific inhibitor LY294002 (Fig. 8).

### PI3K-specific inhibitor LY294002 reversed the protective effects of rh-Aggf1 on neuroinflammation and BBB integrity after SAH

**Fig. 9:**
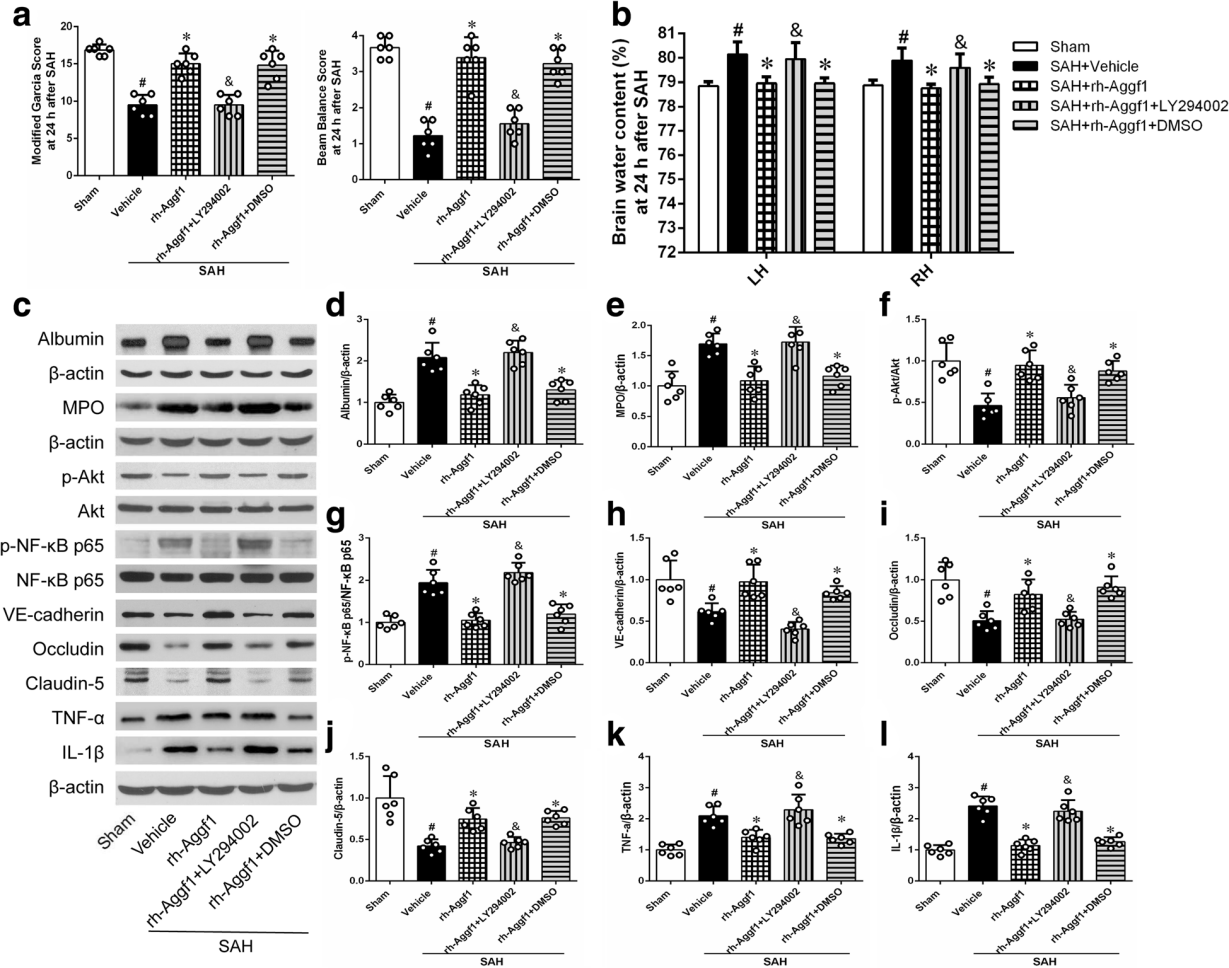
LY294002 reversed the neuroprotection of exogenous Aggf1 after subarachnoid hemorrhage (SAH). **a** Pretreatment of LY294002 abolished the protective effects of rh-Aggf1 on neurological functions. **b**–**e** LY294002 reversed the effects of rh-Aggf1 against brain edema and BBB permeability and inflammatory response (albumin extravasation and MPO expression). **c**, **f**–**l** LY294002 reserved rh-Aggf1 induced changes in protein levels of PI3K, p-Akt, p-NF-κB p65, VE-cadherin, Occludin, claudin-5, TNF-α, and IL-1β in rh-Aggf1-treated rats after SAH. ^#^*P* < 0.05 vs sham; *P < 0.05 vs vehicle; ^&^*P* < 0.05 vs rh-Aggf1 and rh-Aggf1+DMSO. DMSO, dimethyl sulfoxide; LY294002, PI3K-specific inhibitor; MPO, myeloperoxidase

As shown in Fig. 9, LY294002 significantly abolished the protective effects of rh-Aggf1 on neurological functions (Fig. 9a) and brain edema (Fig. 9b) at 24 h after SAH. Consistently, LY294002 reversed the protection of rh-Aggf1 treatment on neuroinflammation and BBB integrity, as the significant increase in albumin leakage and MPO expression was observed at 24 h after SAH (Fig. 9c–e). Moreover, pretreatment with LY294002 sufficiently decreased the expression of p-Akt, VE-cadherin, Occludin, and Claudin-5 with an increase in p-NF-κB p65, TNF-α, and IL-1β expression (Fig. 9c, f–l).

## Discussion

The current study was the first to explore the neuroprotective role of Aggf1 and study the potential mechanisms in a rat model of SAH. Our novel findings are (1) Aggf1 was expressed in the cerebral cortex of rats and upregulated at an early stage following SAH. Endogenous Aggf1 was expressed mainly in the astrocytes, endothelial cells, and microglia; (2) rh-Aggf1 improved neurological deficits and attenuated brain edema and BBB disruption at 24 h after SAH. Furthermore, rh-Aggf1 treatment reduced the numbers of infiltrating neutrophils and activated microglia in the ipsilateral cortex; (3) the knockdown of endogenous Aggf1 by Aggf1 siRNA aggravated neurological impairment, BBB disruption, and inflammatory response after SAH; (4) PI3K/Akt signaling pathway was the underlying mechanism of the neuroprotection provided by Aggf1. Aggf1/PI3K/Akt activation decreased the phosphorylation of downstream NF-κB p65, which was associated with upregulation of endothelial junction proteins levels and downregulation of pro-inflammatory cytokines levels in the brain. The inhibition of PI3K using LY294002 reversed the protective effects of exogenous Aggf1 on brain edema formation, BBB disruption, and neuroinflammation.

BBB disruption, neuroinflammation, and consequent brain edema are characterized as the major pathological changes of brain injury, which result in poor outcome after SAH [4, 28]. Numerous studies showed that Aggf1, a newly identified protein, possesses a strong ability to promote angiogenesis, which was widely expressed in the heart, limb, blood, kidney, and liver under normal or pathological conditions [10, 15, 16, 29, 30]. In the present study, we observed that endogenous Aggf1 expression was increased in the early stage after SAH, and Aggf1 was expressed mainly in the endothelial cells, astrocytes, and microglia. However, this result was different from the previous findings in mice model of carotid artery injury. After carotid artery injury in mice, Aggf1 expression was decreased in the carotid arteries [31, 32]. We supposed that such discrepancy may be due to the difference in animal models and tissue types.

Several studies revealed that Aggf1 exerted anti-inflammatory property and preserved vascular integrity. An in vivo and in vitro study revealed that Aggf1 could inhibit vascular inflammation through suppressing endothelial activation via ERK/NF-κB signaling pathway in the hindlimb ischemia model, while the knockdown of Aggf1 increased the expression of these pro-inflammatory molecules and promoted the monocyte-endothelial cells interaction [14]. During myocardial ischemia-reperfusion injury, Aggf1 was proven to decrease the release of inflammatory molecules and decrease infarct size [13]. Recently, a compelling study showed that Aggf1 knockout mice exhibited significantly impaired vascular development, frequent hemorrhages, and increased vascular permeability in embryos and yolk sacs, which could be rescued by exogenous Aggf1 treatment, suggesting that Aggf1 has a critical role in the maintenance of vascular integrity [16]. Consistent with the previous findings, our observations revealed that treatment with rh-Aggf1 could reduce brain edema and BBB disruption, inhibit neutrophil infiltration and microglia activation, and improve neurological function at 24 h after SAH in rats. Whereas, knockdown of endogenous Aggf1 worsened neurological deficits, BBB breakdown, and inflammatory response, which indicated that Aggf1 could attenuate neuroinflammation and BBB disruption following SAH.

The NF-κB signaling pathway is involved in regulating inflammation and BBB integrity in cerebral ischemia and neurodegenerative diseases [19, 33, 34]. The PI3K/Akt signaling is well known as a major upstream element of the NF-κB signaling pathway. A recent study demonstrated that Aggf1 could activate the PI3K/AKT signaling pathway important for angiogenesis and regulate phosphorylation of VE-cadherin important for maintenance of myocardial vascular integrity, which indicated that PI3K/AKT signaling pathway was a molecular downstream of Aggf1 action [16]. In the present study, our results showed that administration of exogenous Aggf1 significantly upregulated the expression of PI3K and p-Akt; decreased p-NF-κB p65, TNF-α, and IL-1β; and reduced neutrophil infiltration and microglia activation, thus improved neurological deficits after SAH. Meanwhile, rh-Aggf1 treatment resulted in an increase of Occludin, Claudin-5, and VE-cadherin and preserved BBB integrity. In contrast, these effects of rh-Aggf1 were reversed by PI3K-specific inhibitor LY294002, due to suppressing AKT phosphorylation and activating downstream NF-κB, subsequently increasing inflammatory molecules and decreasing tight junction proteins in protein level. Taken together, our results suggested that activation of PI3K/AKT/NF-κB signaling pathway underlays the Aggf1-mediated neuroprotection after SAH.

Up to now, the specific intermediate molecules have not been identified between Aggf1 and PI3K. However, some evidences suggested that a unique receptor for Aggf1 might exist because (1) Aggf1 began to redistribute by moving towards the cell periphery and was secreted outside the endothelial cells when angiogenesis was initiated in an in vitro [6]; (2) purified Aggf1 was able to bind to cultured endothelial cells in calcein allantoic membrane-based cell adhesion assays [6]. Future studies are warranted to elucidate whether there are any intermediate molecules between Aggf1 and PI3K/Akt/NF-κB signaling pathway.

Previous studies reported SAH-induced hippocampal damage at an early stage, which was associated with the subsequent cognitive and memory impairment [35, 36]. In our study, rh-Aggf1 improved long-term neurological function in terms of foot-fault test and Morris water maze trial, which may be related to the attenuation of brain injury.

There were some limitations in our study. Only intravenous route and one-time point (1 h after SAH) of rh-Aggf1 administration was applied in the present study, which did not evaluate the best route of delivery and the potential therapeutic window of rh-Aggf1 treatment for SAH. Aggf1 exerts multiple protective properties against various pathological processes, including anti-inflammation, anti-apoptosis, and activation of autophagy [12, 13]. In the present study, we only investigated the neuroprotective effects of Aggf1 on neuroinflammation and BBB integrity after SAH in rats. Hence, we can not rule out the possibility that Aggf1-mediated other effects may play protective roles in brain injury after SAH. Future studies are needed to further explore the other functions as well as its possible underlying mechanisms.

## Conclusion

In summary, our findings suggested that administration of exogenous Aggf1 could improve both short- and long-term neurological impairments and attenuate inflammatory response and BBB disruption after SAH in rats. The neuroprotective effects of Aggf1 were mediated at least via PI3K/Akt/NF-κB signaling pathway. Thus, exogenous Aggf1 might offer a promising strategy against neuroinflammation in patients with SAH.

## Additional files

Additional file 1: **Table S1.** Numbers of animals used in each group. (DOCX 19 kb)

Additional file 2: **Figure S1.** Subarachnoid hemorrhage (SAH). (A) Representative images in sham and SAH groups. Subarachnoid blood clots were mainly present around the circle of Willis at 24 h post-SAH. (B) SAH grade scores of all SAH groups at 24 h post-SAH. Aggf1, Angiogenic factor with G patch, and FHA domains 1; DMSO, dimethyl sulfoxide; Scr siRNA, scrambled siRNA; LY294002, PI3K-specific inhibitor. (TIF 442 kb)

## Abbreviations

Aggf1: Angiogenic factor with G patch and FHA domains 1
BBB: Blood-brain barrier
DMSO: Dimethyl sulfoxide
EB: Evans blue
EBI: Early brain injury
GFAP: Glial fibrillary acidic protein
Iba-1: Ionized calcium-binding adaptor molecule 1
IHC: Immunohistochemistry
MPO: Myeloperoxidase
NS: Normal saline
SAH: Subarachnoid hemorrhage
VEGFA: Vascular endothelial growth factor A
vWF: Von Willebrand factor
