# Supplementary material for: Aggf1 attenuates neuroinflammation and BBB disruption via PI3K/Akt/NF-κB pathway after subarachnoid hemorrhage in rats
Source: J Neuroinflammation. 2018 Jun 9;15:178. doi: 10.1186/s12974-018-1211-8 (PMC5994242; doi:10.1186/s12974-018-1211-8)
Supplement: Supplementary file 1 — Table S1. Numbers of animals used in each group. (DOCX 19 kb) [file 12974_2018_1211_MOESM1_ESM.docx]

**Additional file 1**

**Table S1** Numbers of animals used in each group.

| Experimental Group | Neurobehavioral test | | EB | WB | IHC | Death | Exclusion | Subtotal |
| --- | --- | --- | --- | --- | --- | --- | --- | --- |
|  | Brain edema | Long-term |  |  |  |  |  |  |
| Sham | 6 | 8 | 6 | 6 | 3 | 0 | 0 | 29 |
| SAH (3h, 6h, 12h, 24h, 72h) |  |  |  | 30 | 3 | 6 | 2 | 41 |
| SAH+Vehicle | 6 | 8 | 6 | 6 | 3 | 6 | 2 | 37 |
| SAH+rh-Aggf1 (1 µg/rat) | 6 |  |  |  |  | 1 | 1 | 8 |
| SAH+rh-Aggf1 (3 µg/rat) | 6 | 8 | 6 | 6 | 3 | 5 | 2 | 36 |
| SAH+rh-Aggf1 (9 µg/rat) | 6 |  |  |  |  | 2 | 1 | 9 |
| SAH+Aggf1 siRNA | 6 |  | 6 | 6 |  | 3 | 1 | 22 |
| SAH+Scr siRNA | 6 |  | 6 | 6 |  | 4 | 1 | 23 |
| SAH+rh-Aggf1+LY294002 | 6 |  |  | 6 | 3 | 4 | 1 | 20 |
| SAH+rh-Aggf1+DMSO | 6 |  |  | 6 |  | 4 | 2 | 18 |
| Naive+Aggf1 siRNA |  |  |  | 3 |  | 0 | 0 | 3 |
| Naive+Scr siRNA |  |  |  | 3 |  | 0 | 0 | 3 |
| Total | 54 | 24 | 30 | 78 | 15 | 35 | 13 | 249 |

Aggf1, Angiogenic factor with G patch and FHA domains 1; DMSO, Dimethyl sulfoxide; EB, Evans blue; IHC, Immunohistochemistry; LY294002, PI3K inhibitor; NS, normal saline; SAH, subarachnoid hemorrhage; Scr siRNA, Scrambled siRNA; WB, Western blot.
