# Supplementary figures and images for: Aggf1 attenuates neuroinflammation and BBB disruption via PI3K/Akt/NF-κB pathway after subarachnoid hemorrhage in rats
Source: J Neuroinflammation. 2018 Jun 9;15:178. doi: 10.1186/s12974-018-1211-8 (PMC5994242; doi:10.1186/s12974-018-1211-8)

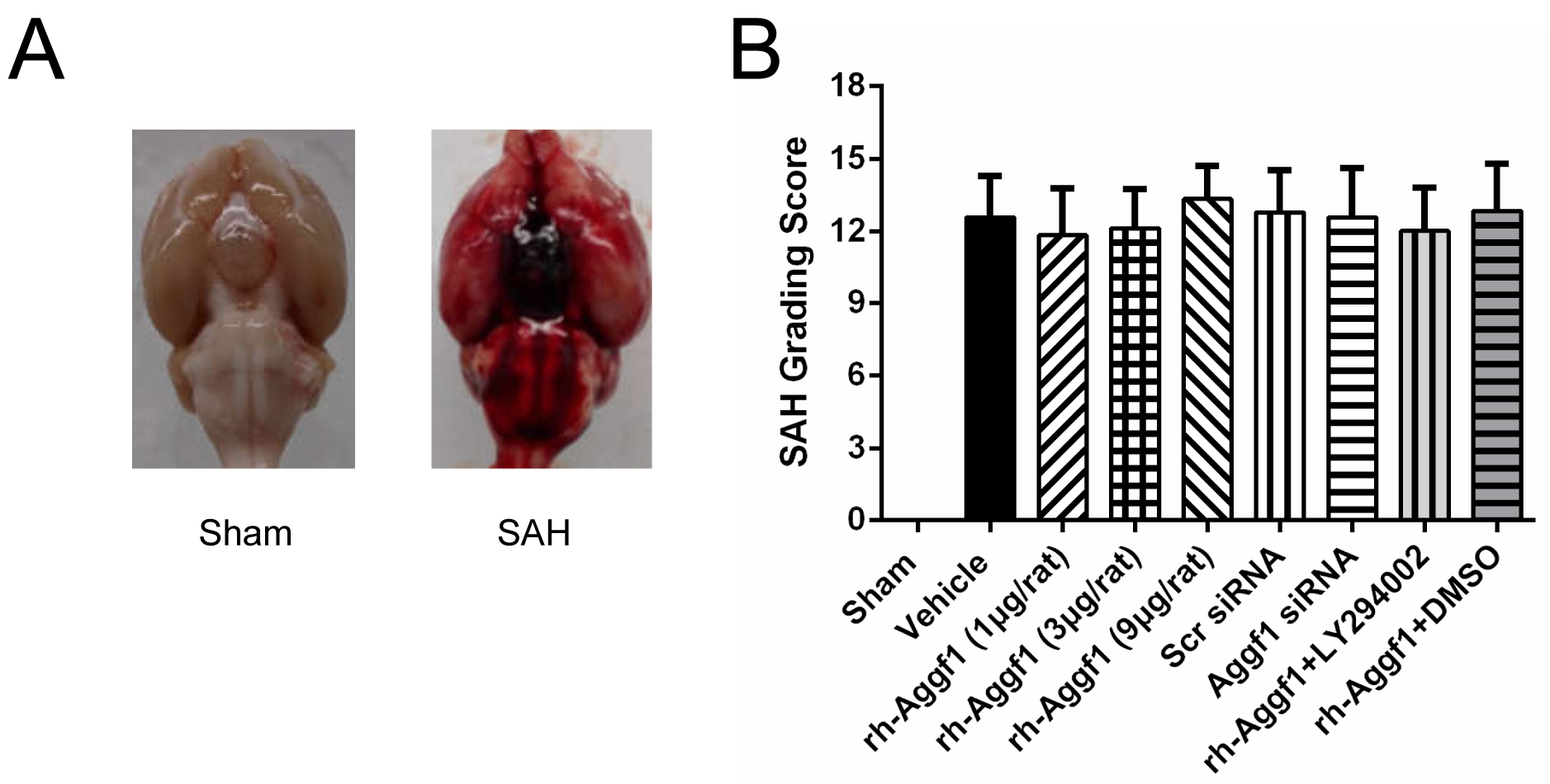

Supplement: Supplementary file 2 — Figure S1. Subarachnoid hemorrhage (SAH). (A) Representative images in sham and SAH groups. Subarachnoid blood clots were mainly present around the circle of Willis at 24 h post-SAH. (B) SAH grade scores of all SAH groups at 24 h post-SAH. Aggf1, Angiogenic factor with G patch, and FHA domains 1; DMSO, dimethyl sulfoxide; Scr siRNA, scrambled siRNA; LY294002, PI3K-specific inhibitor. (TIF 442 kb) [file 12974_2018_1211_MOESM2_ESM.tif]
